# Intraoperative Intercostal Nerve Cryoneurolysis for Post-Thoracotomy Pain Control: A Case Discussion

**DOI:** 10.31480/2330-4871/159

**Published:** 2022-09-12

**Authors:** Xiaowei Lu, Ning Miao, Sandra Orfgen, Andrew Mannes

**Affiliations:** Department of Perioperative Medicine, Clinical Center, NIH, USA

## Introduction

Mesenchymal chondrosarcoma (MCS) is an extremely rare, often high-grade aggressive malignancy. MCS has a greater incidence in young adults, and approximately two-thirds of cases affect bone (spine, ribs or jaw) and soft tissues [1–3].

MCS arising from the rib is clinically managed with a thoracotomy for the resection of tumors. However, chest wall surgery is frequently associated with severe, postoperative pain. Inadequate pain control in this period increases the risk of chronic post-thoracotomy pain syndrome and postoperative complications, decreases physical function, and worsens quality of life [4–6].

Management of post-thoracotomy pain includes systemic medication, neuraxial injections, regional nerve blocks and interventional techniques. Here, we describe the use of intercostal nerve cryoneurolysis (IC) using a probe that creates a frigid surface which is placed adjacent to the intercostal nerves and the tissue. The temperature of the probe tip decreased to between −50 °C to −70 °C. The nerve axon degenerates interrupting the painful stimuli, but leaves the fibrous outer neural structures intact [6,7].

We present a 33-year-old patient diagnosed with a large, intrathoracic mesenchymal chondrosarcoma and extensive arm sensory and motor involvement was referred to our hospital for tumor resection. At the completion of surgery, cryoneurolysis was utilized for post-operative pain management.

## Case Presentation

**Figure 1:**
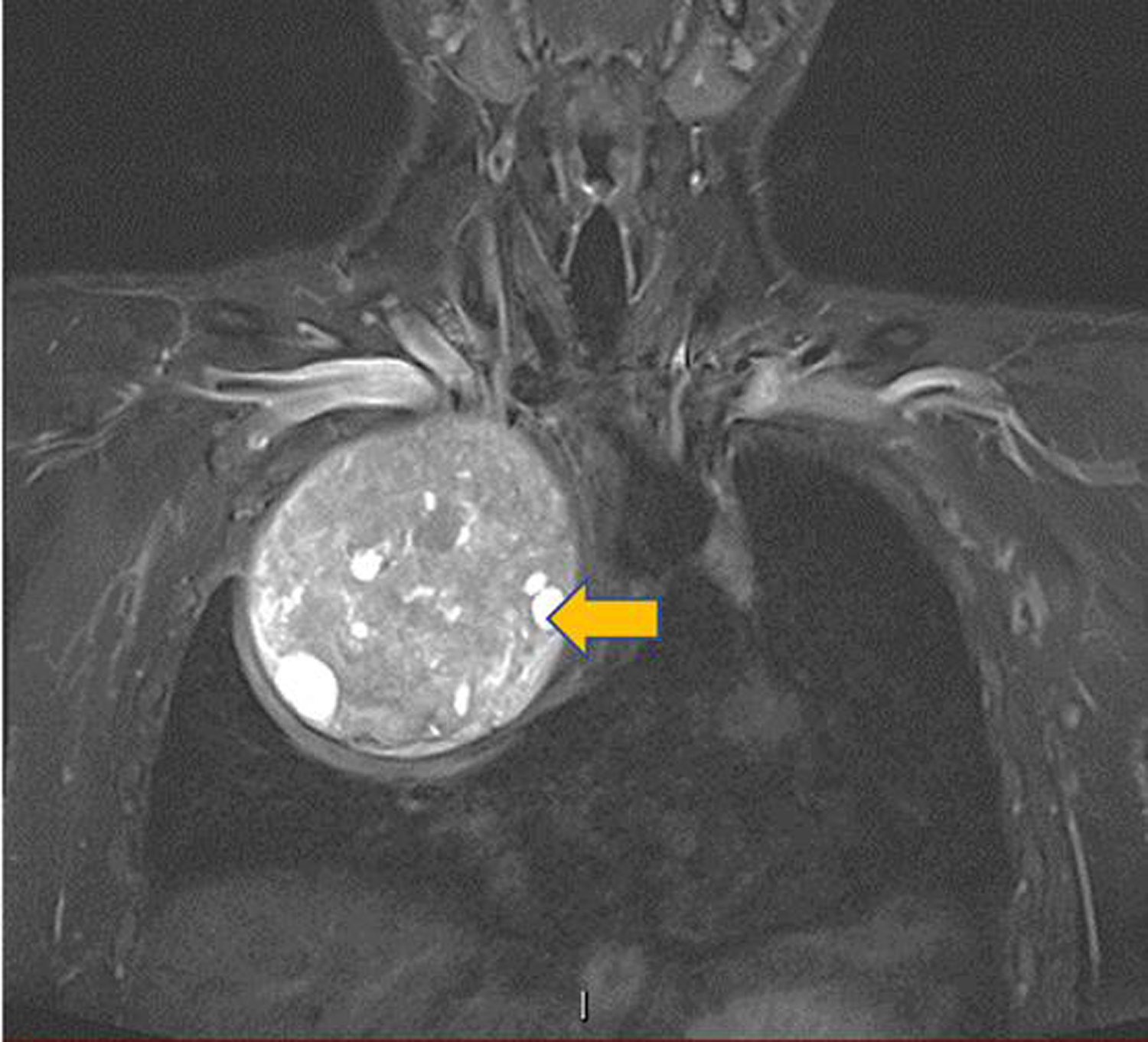

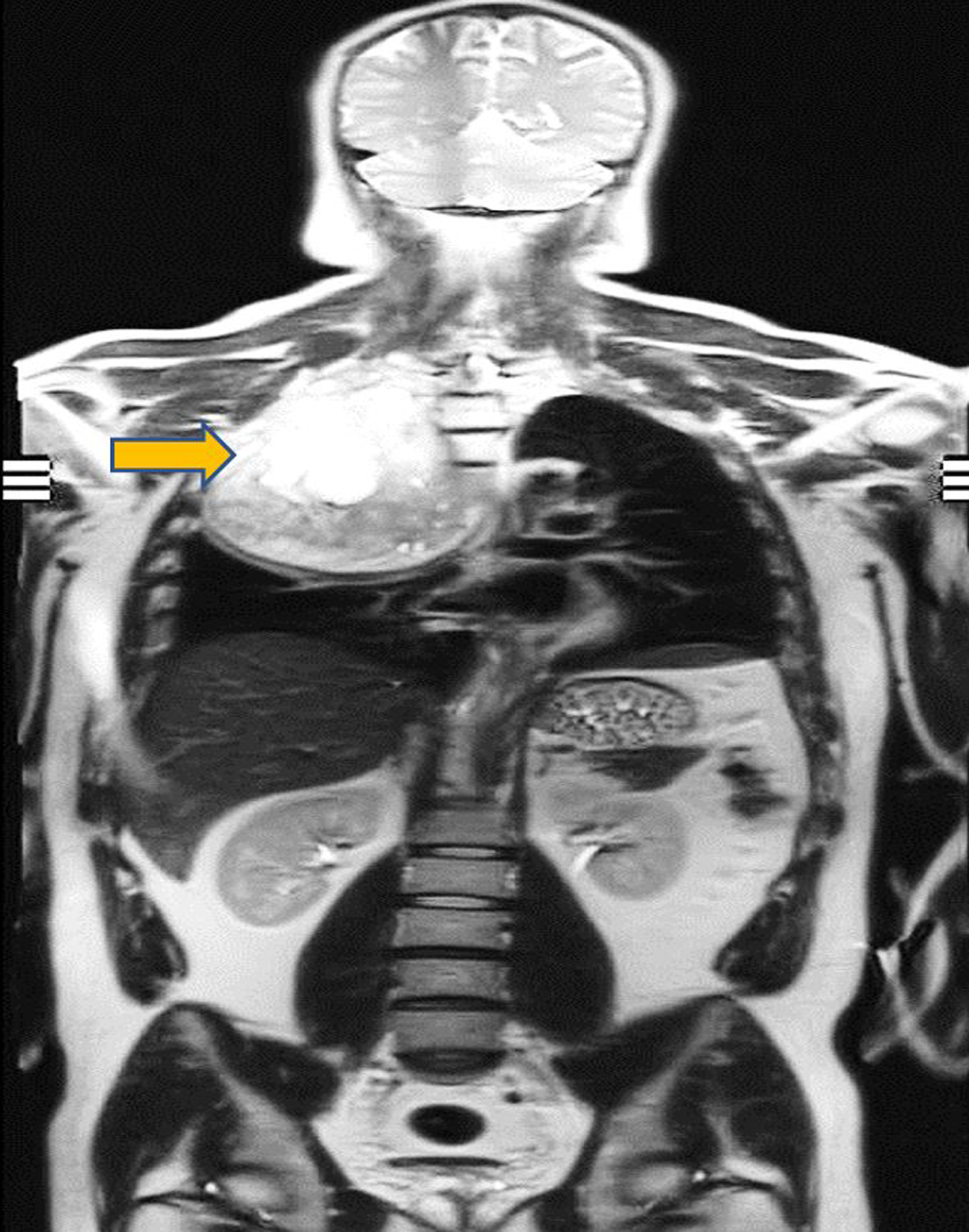
Preoperative Chest MRI image: Heterogeneously enhancing mass (arrows) in the right thoracic inlets and lung apex (13.4 cm × 12.3 cm).

A 33-year-old man presented with right chest and arm pain, right arm sensory and motor deficits along with the ulnar nerve distribution, and right arm anhidrosis. Imaging was remarkable for a 13.4 cm × 12.3 cm heterogeneously enhancing mass from the right 2^nd^ rib growing into the right thoracic inlet and right lung apex. The tumor caused normal lung parenchymal deviation with extensive right-sided neoplastic neuroforaminal involvement and brachial plexus compression (Figure 1). A biopsy of the mass was reported as mesenchymal chondrosarcoma. After chemotherapy and radiation therapy, some of his symptoms, including right arm pain improved and the total tumor volume was slightly reduced. The patient was not taking pain medication at the time he was referred to our hospital for neuro/thoracic surgical consultations for possible tumor resection.

On admission, the patient stated he has been doing well, and denied shortness of breath, dyspnea on exertion, nausea, vomiting or worsening right arm sensory/motor dysfunction. His physical exam, laboratory studies, echocardiogram, electrocardiogram (EKG), chest X-ray (CXR) and pulmonary function testing (PFT), chest computed tomography (CT) scan and magnetic resonance imaging (MRI) were all stable.

### Surgical plans and procedures

**Figure 2:**
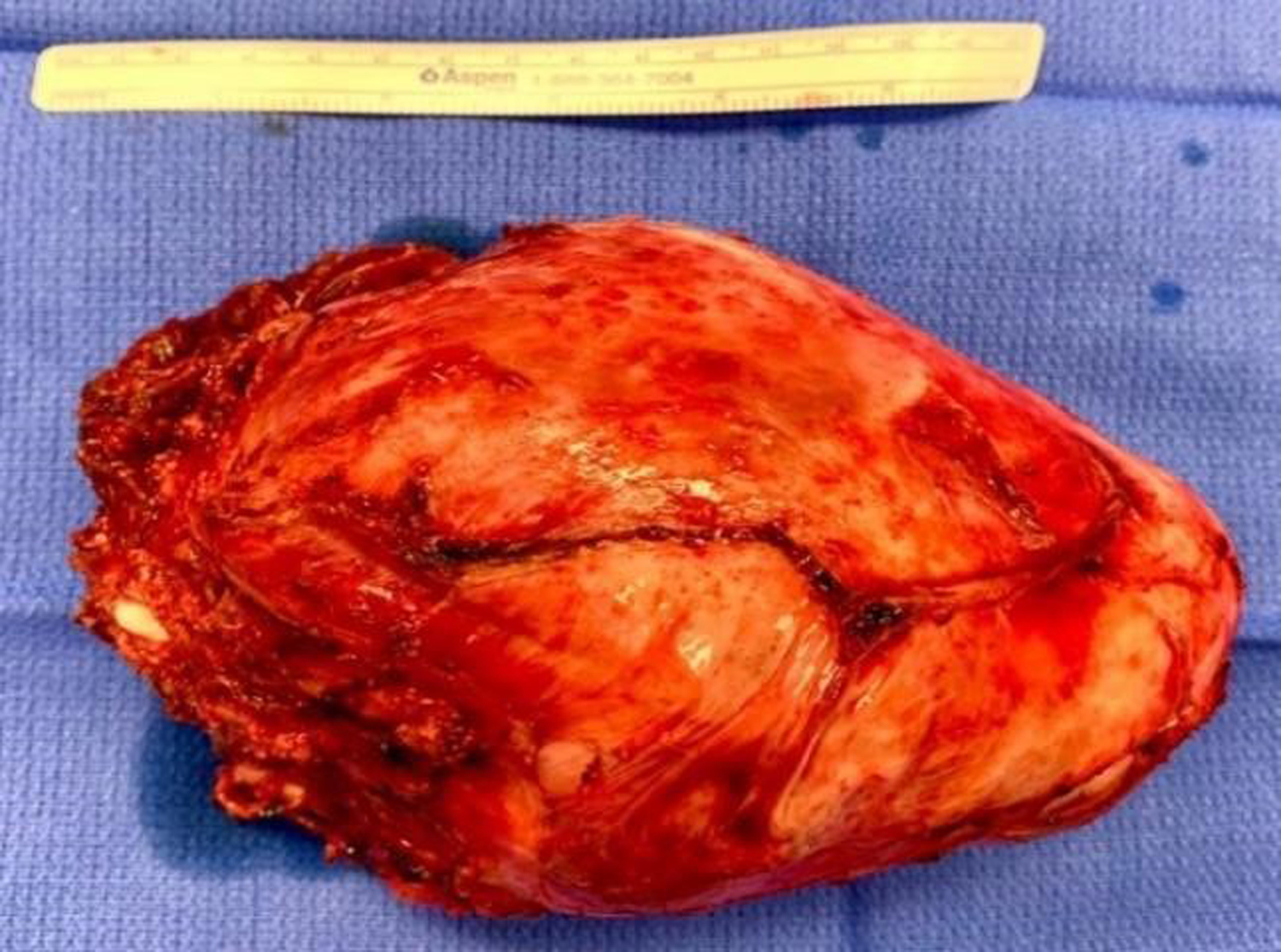

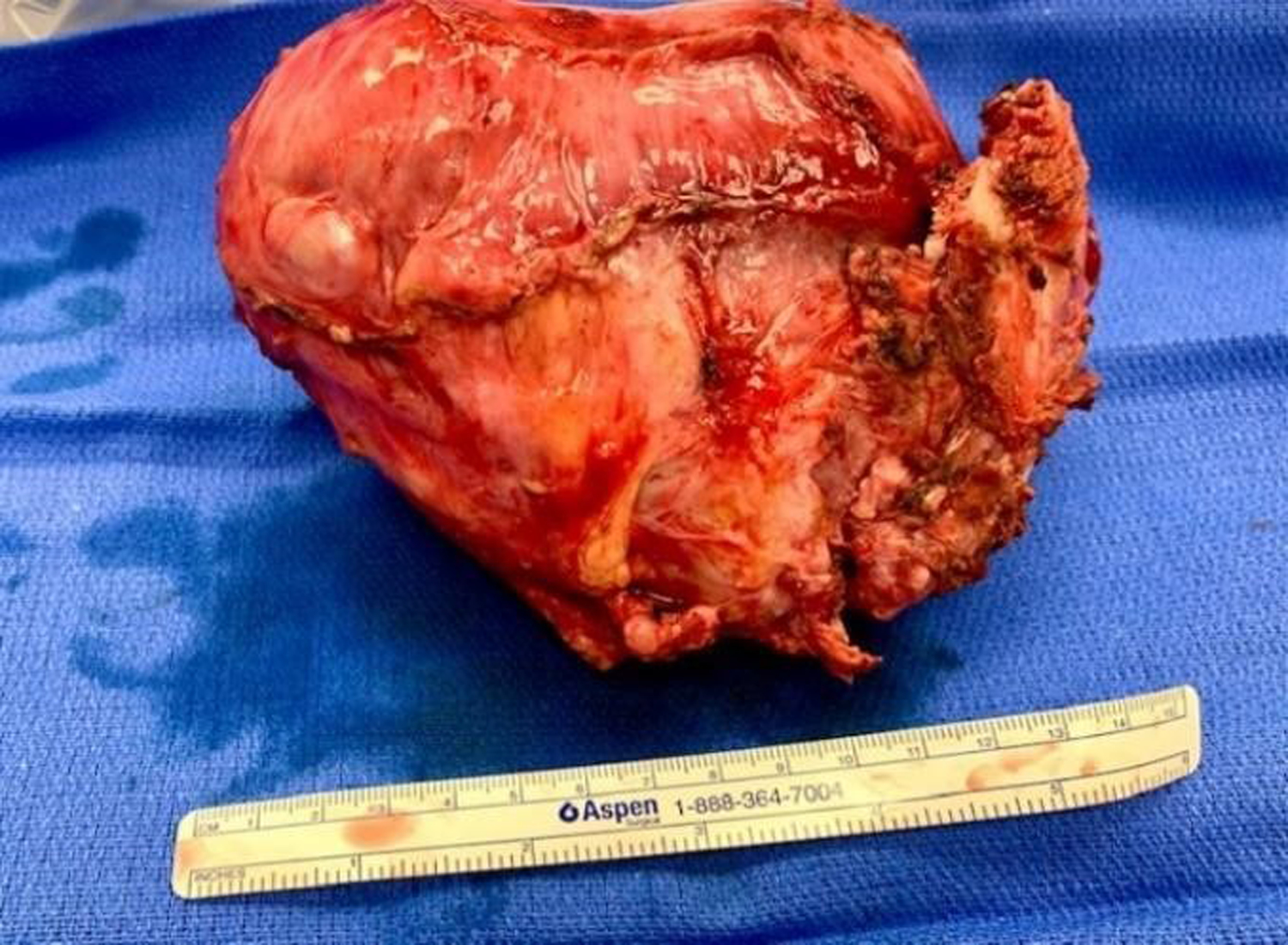
En bloc resection of mesenchymal chondrosarcoma from inside of right chest.

To start the case, the neurosurgeon placed the patient in the prone position to facilitate the dorsal approach to the right of T1–T4 spinous processes and corresponding vertebral bodies. At the level of T2 and T3, the transverse processes were transected, ribs disarticulated and the nerve roots ligated. The patient was then repositioned into the left decubitus position for the chest wall tumor resection including en bloc resection of a portion of T2 and T3 transverse processes (no reconstruction) (Figure 2).

**Figure 3:**
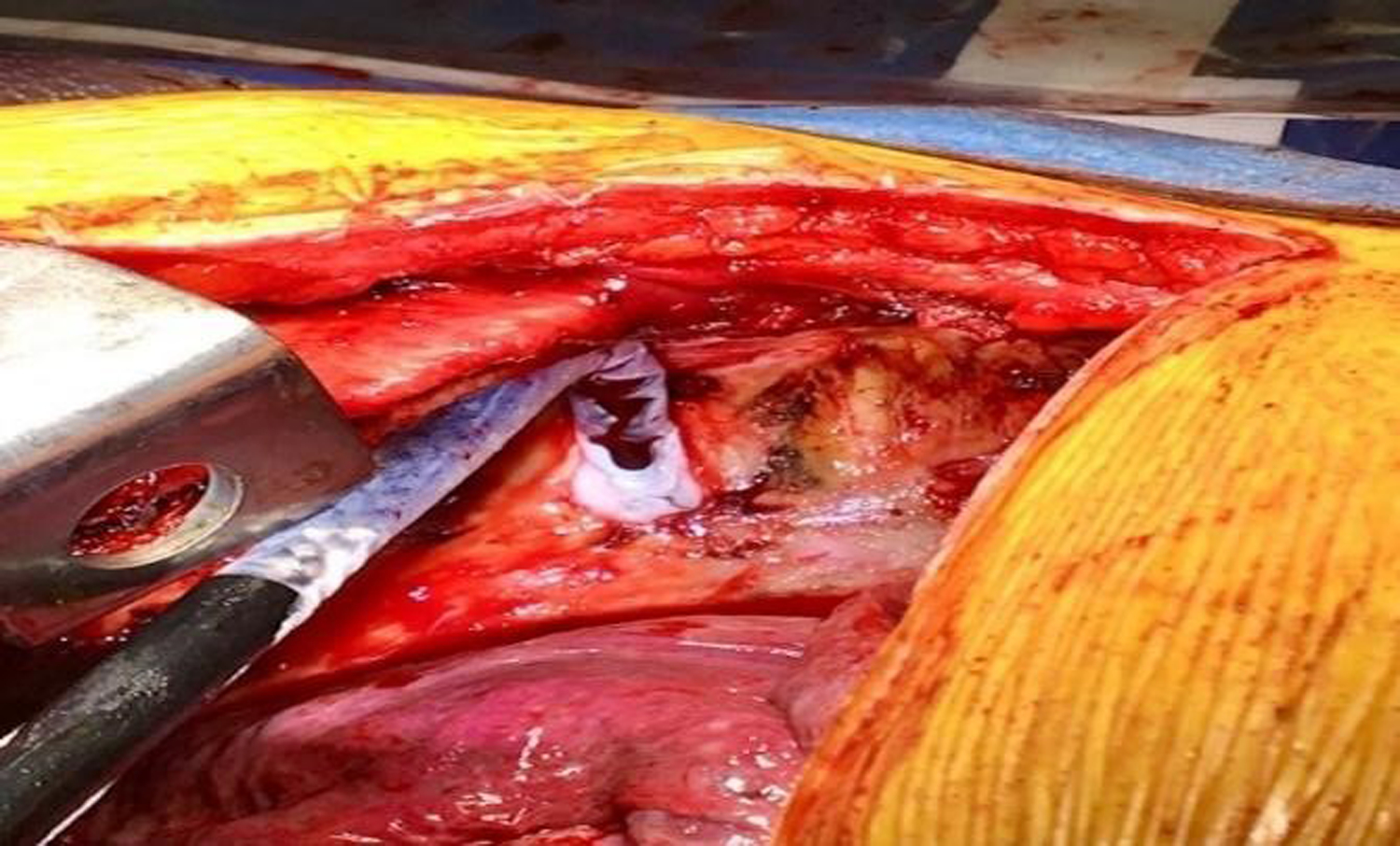

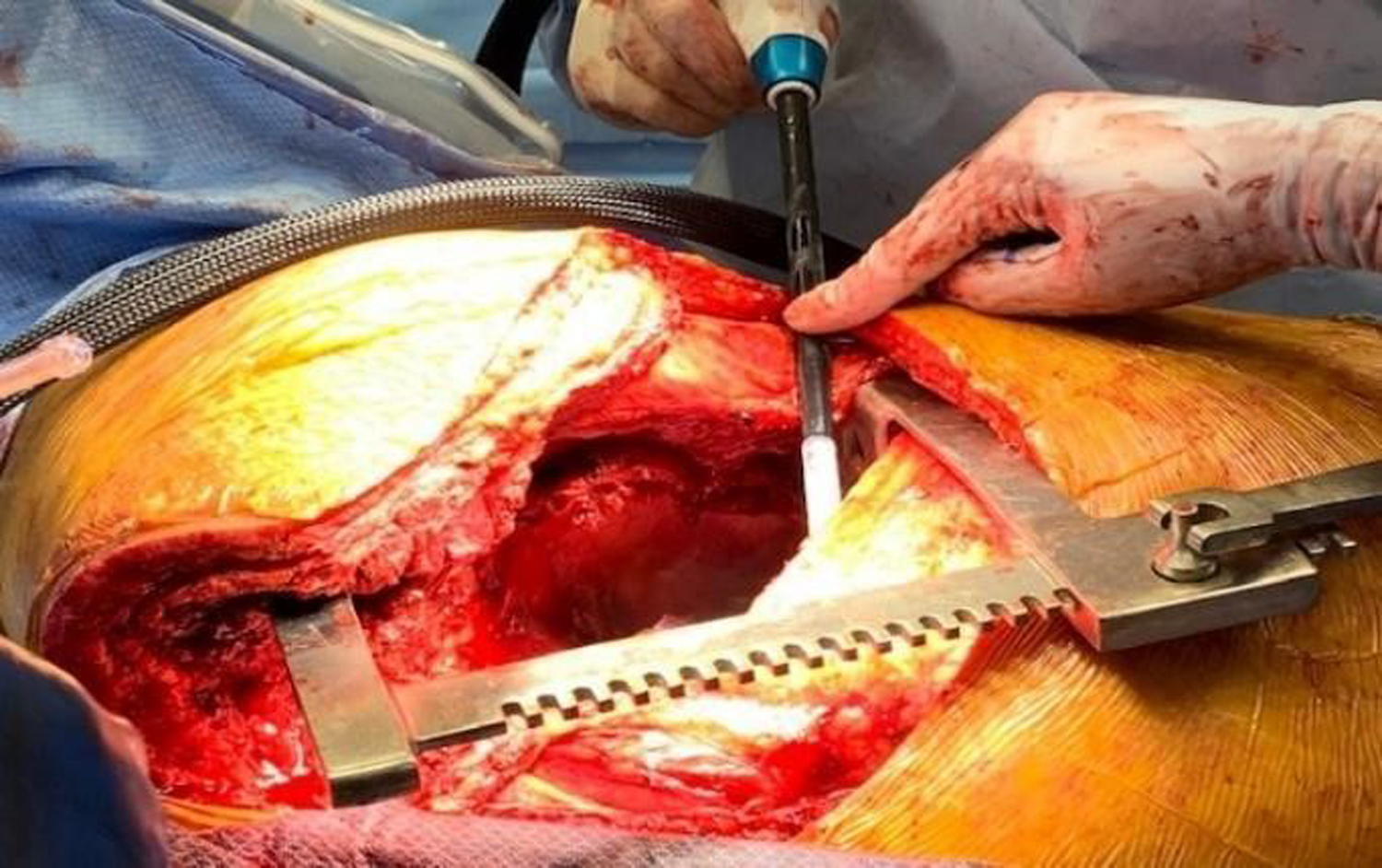
Cryoneurolysis placement (arrow) and iceball formation on the intercostal nerve.

After the tumor was resected, the thoracic surgeons performed intercostal cryoneurolysis (IC) of the three-level of ribs encompassing the surgical incision. The cryoprobe (cryoICE TM, Atricure Inc., Ohio) was placed into the thoracic cavity under direct visualization. It touched the membranous portion of the right fourth through sixth intercostal space, 4 cm away from the base of the spine to avoid iatrogenic injury to the sympathetic ganglia chain (Figure 3). Each nerve was frozen for 120 sec with a tip temperature between −50 °C to −70 °C. After the cryoneurolysis, the thoracic surgeon re-inspected the chest, established hemostasis and closed the surgical wound. The patient tolerated the surgery and remained stable throughout the entire 8-hour procedure.

### Anesthesia management

The patient was taken to the operating room after pre-medication with IV midazolam. American Society of Anesthesiologists (ASA) standard monitor were placed. The patient was pre-oxygenated with 100% O_2_ and induced with IV fentanyl, propofol and rocuronium. A 7.5 reinforced endotracheal tube (ETT) was placed smoothly and anesthesia was maintained with air, O_2_, sevoflurane, fentanyl and intermittent rocuronium boluses. An arterial line was inserted in the radial artery, 16G IV catheter was placed in an antecubital vein and the bladder was catheterized. The patient was placed in the prone position for the neurosurgery procedure and vital signs, ETT, IV flow and arterial waveform were rechecked after positioning.

Subsequently, the patient was repositioned back into supine position for the thoracic and IC procedure. The single lumen ETT was exchanged uneventfully for a #37 left double lumen tube (DLT), and one lung ventilation was applied for the procedure. The surgical debulking, dissecting and en bloc resection of the apical mass resulted in a 1250 ml blood loss. Fluid replacement consisted of 1000 ml lactated ringers and 400 ml 25% albumin. Calcium chloride 1000 mg and magnesium sulfate 2000 mg were administered. An ABG drawn at the end of surgery showed normal electrolytes and Hb 7 g/dl, but no intraoperative blood transfusion was required. At the end of the surgery, the patient was extubated and transferred to Intensive Care Unit (ICU) for post-operative monitoring and care.

### Postoperative recovery

Postoperatively, the patient was transfused three units of red blood cells (RBC) which stabilized his hemodynamic status for his symptomatic anemia. He reported 2–3/10 pain corresponding to his right posterior chest wall where the nerve roots of T2 and T3 were ligated that was adequately treated with intermittent IV hydromorphone boluses. However, the chest wall area which corresponds to the T4–T6 cryoneurolysis remained pain free. On postoperative day 3, he required only 5 mg oral oxycodone per day for pain control. He had no difficulty with pulmonary physiotherapy reaching incentive spirometry values > 50% of predicted and he was able to produce a strong cough. His right arm grip, finger extension, wrist flexion/extension, and biceps, triceps motor function were normal but reduced in the deltoid muscle. He experienced decreased sensation to light touch over the medial upper arm and chest above the T5 dermatome. The patient was discharged home uneventfully on postoperative day 5 with a prescription for oxycodone 5 mg, ibuprofen 600 mg, and acetaminophen 650 mg, all to be taken up to 3 times per day as needed. Upon discharge from the hospital, the patient reported excellent pain relief.

## Discussion

### Mesenchymal chondrosarcoma (MCS)

MCS, an extremely rare malignant type of chondrosarcoma accounting for approximately 5–10% of all cases was first described in the medical literature in 1959. Most MCS occurs with greater frequency in young adults and affects the bones, especially the spine, ribs or jaws. The remaining cases occur extra-skeletally, such as in the CNS, muscle and fat. Unlike other types of malignant chondrosarcoma, which have a tendency to grow more slowly and rarely develop metastases, MCS is a fast growing tumor that spreads more frequently [1–3,8,9].

The symptoms of MCS are variable and nonspecific depending upon the exact location and progression of the tumor. Many patients may develop pain, numbness and swelling in the affected areas. The exact cause of mesenchymal chondrosarcoma is unknown, and it is often diagnosed when it causes symptoms related to the physical location of the tumor. Although images can help show the tumor’s location, size and its relation to adjacent tissues, they are not specific. A biopsy is recommended to identify the histological cell type that diagnoses the tumor and differentiates it from other types of sarcoma [3,9].

The initial treatment for a MCS is surgery and/or chemotherapy. Usually, chemotherapy will be given first to reduce the tumor size and facilitate surgical removal of the entire tumor and adjacent affected tissue. Surgery with wide radical excision is the mainstay of treatment with a better survival. Radiation can be considered as standard practice for larger tumors [2,8].

### Conventional pain control for thoracotomy

An open thoracotomy can cause significant and even chronic postoperative pain which has remained a significant challenge to medical professionals. Inadequate postoperative pain control may increase lung infection, atelectasis, hypoxia or respiratory failure. The most effective techniques for post-thoracotomy analgesia include continuous thoracic epidural analgesia or paravertebral blocks. However, those techniques possess contraindication in some patients (e.g. genetic disease such as Von Hippo Lindau disease, patients with a spine injury or coagulopathy). A variety of other multimodal regimens include erector spinae block and intercostal nerve block, systemic narcotics IV PCA provide short-term analgesia, and the analgesic duration is typically insufficient for the entire recovery period [5,6,10].

### Intercostal cryoneurolysis (IC)

IC is another treatment for post-thoracotomy pain control, particularly for patients with refractory chest wall symptoms or thoracic surgeries where conventional therapies failed. This technique provides long-acting intercostal nerve blockade that outlasts injections and catheter-based delivery systems, providing analgesia throughout the postoperative period associated with the most severe pain.

Knowledge of thoracic nerve distribution and nerve anatomy is pivotal to understanding the IC’s use for pain control with thoracic surgery. The intercostal nerves arise from the thoracic spinal nerves that comprise the sensory and motor bundle derived from the spinal cord. Innervation of these nerves controls the contraction of chest wall muscles, as well as provides sensory input of the skin and parietal pleura. Thus, damage to the internal wall of the thoracic cavity can be felt as a sharp pain localized in the injured region [11].

**Figure 4:**
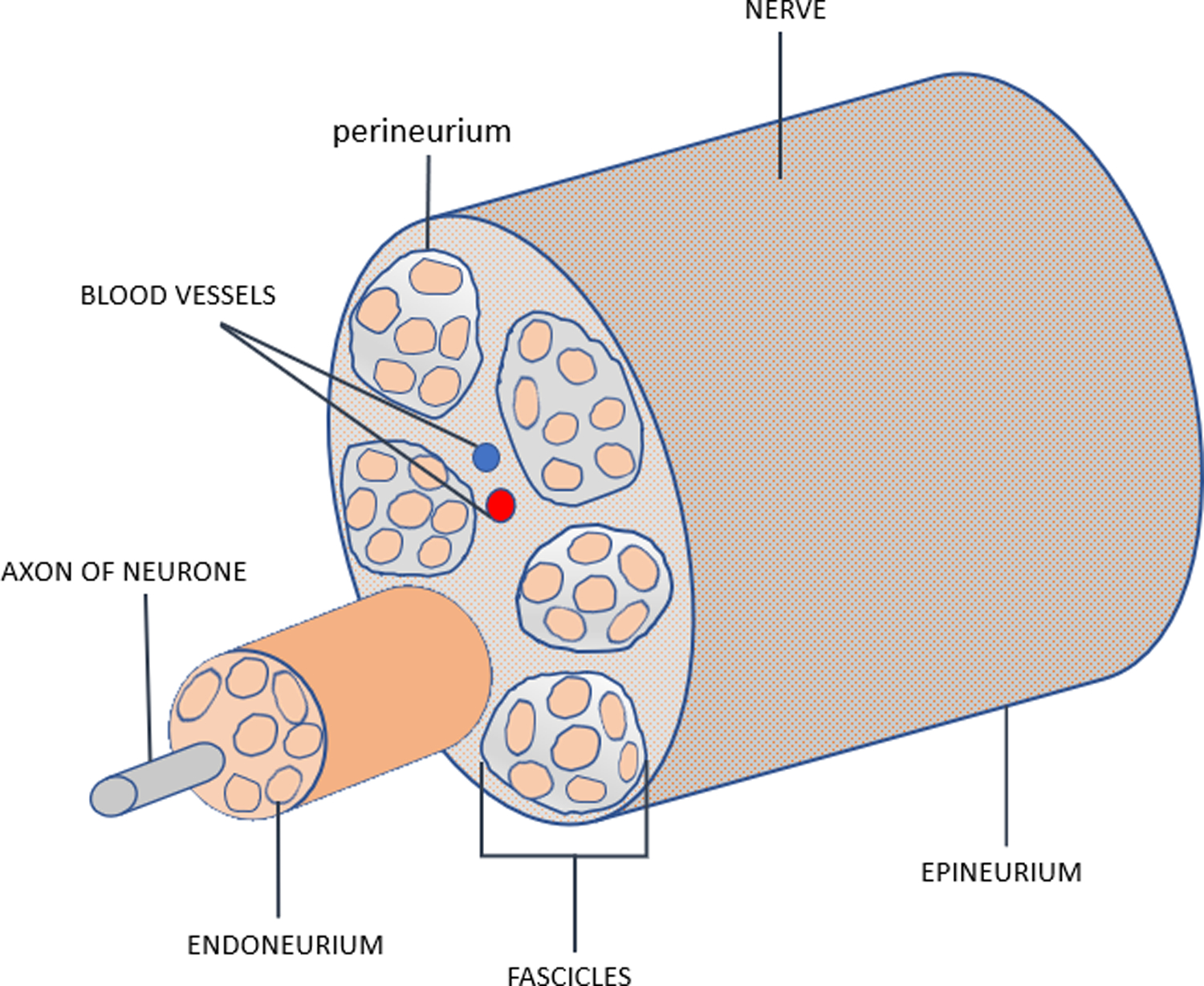
Peripheral nerve anatomical structure.

The intercostal nerves (Figure 4) originate from T1–11 ventral rami and innervate the intercostal muscles and provide sensory input from the skin, muscle and parietal pleura. Each nerve axon is surrounded by endoneurium, a layer of connective tissue, and are bundled together into fascicles. Each fascicle is wrapped in a layer of connective tissue called the perineurium and the entire nerve is wrapped in a layer of connective tissue called the epineurium. The different tissue layers play a role in protecting neurons from chemical or mechanical injury (perineurium), and facilitate the blood and lymph flow (epineurium and endoneurium) [12,13].

There are three major types of nerve injury reacting to the cold temperature application: neuropraxia, axonotmesis, and neurotmesis. At the temperature of 10 °C to −20 °C, neuropraxia develops with little or no injury to the nerve and is limited to the epineurium and neural recovery time varies [14]. When the temperature dropped to −20 °C - to −100 °C, the axonotmesis is generated through Wallerian degeneration-a process of a degeneration at the axon distal to the injury. This degeneration effectively inhibits the nerve signal transmission associated with pain sensation [14]. Within this temperature range, the three fibrous layers (epineurium, perineurium, and endoneurium) remain intact. The degenerative nerves then regrow at the rate of 1–2 mm/day from the injured point distally. This reversible degeneration and slow recovery make the pain control last weeks to months. A temperature lower than −100 °C on the nerve bundle injures the endoneurium resulting in neurotmesis- a process that affects axon regrowth permanently [13].

During the thoracic surgery, the cryoprobe has nitrous oxide within the probe expended forming a low temperature ice ball (−50 °C to −70 °C) at the tip. The active surface is directly applied to the intercostal space and freezes the intercostal nerves to produce the prolonged desensitization. This degenerates the enclosed nerve axons but leaves the fibrous outer neural structures intact, resulting in numbness distal to the nerves (due to interrupting pain signaling transmission). Sensation will return as the nerve axons regenerate within the intact nerve sheath and the rate and extent of axon regeneration is a function of the time and intensity of the initial cold application. Typically, the intercostal axon regenerates and normal nerve function returns after one to three months [5,15–17]. The other effects of cryoneurolysis have also been studied. There is no permanent motor nerve injury [18,19] and proprioception is similarly decreased at an acceptable level [20].

Cryoneurolysis has been used for 50 years but there is a renewed interest in acute and chronic pain control as the technology has been updated. Unlike other ablative techniques, it has a good safety profile with the adjacent structures since blood vessels and bone are resistant to freeze injury [21]. General contraindications to cryoneurolysis include bleeding, anticoagulation, infection and immunosuppression. Other disease-specific contraindications include cold urticaria, Raynaud’s disease, cryofibrinogenemia, cryoglobulinemia and cold paroxysmal hemoglobinuria [22].

### Anesthesia management

This case presented many anesthesia challenges. When the patient is placed in the prone position for surgical procedures, the anesthesia provider must optimize care to avoid the problems with the airway management (potential displaced ETT), ventilation, hemodynamic instability, position-related injuries (e.g. postoperative visual loss-POVL, peripheral nerve injury (PNI), and spinal cord injury) [23]. During one-lung ventilation for thoracotomy, anesthesia providers should employ a protective ventilation strategy, which includes maintenance of low tidal volume (TV), low airway pressure, low positive end expiratory pressure (PEEP) and keep minimum oxygen (O_2_) concentrations to avoid lung injury. Anesthesia providers must be cognizant of the differences in left and right lung anatomy and function, troubleshoot and manage the possible intraoperative malpositioning of the DLT, and the desaturation related to one lung ventilation [24]. The fluid regimen should be individualized to optimize cardiac output (CO) and O_2_ delivery. Excessive fluid administration (i.e. > 3 L in the 24 hours of the perioperative period) is associated with acute lung injury and delayed recovery after open thoracic surgery [10].

Postoperatively, this patient reported pain in the area innervated by T2 and T3 corresponding to surgically ligated intercostal 2 and 3 nerve roots (with no cryoneurolysis), but no pain in the right chest wall dermatomes innervated by T4–T6 (site of cryoneurolysis). He was discharged home on postoperative day 5 with good pain control and no significant complications from surgery or cryoneurolysis. While we report this case study for a single procedure, when compared to our typical post-thoracotomy patient, he had reduced opioid requirements. Given the current opioid crisis, the emphasis on minimization of opioid prescriptions is another reason IC should be considered as an important adjunct for control of post-thoracotomy pain. This is supported by other studies that have confirmed that IC can decrease post-thoracotomy opioid usage, improve pulmonary mechanics, shorten hospital stay, provide long-acting postoperative pain controland lower the risks of complications with a single application [17,25,26].

## Conclusion

Mesenchymal chondrosarcoma is an extremely rare and aggressive malignancy that affects bone and soft tissues, especially the spine, ribs or jaws. Surgical intervention is indicated for managing MCS in the chest. However, a thoracotomy is reported to be one of the most painful operations. Although there are a variety of pain control strategies, they are all typically very short-termed and associated with other limitations and complications. IC provides a profound and durable analgesic response reducing opioid usage and facilitating faster recovery of pulmonary function and long-acting regional nerve blockade. Thus, this technique has been shown to be another valuable tool for post-thoracotomy pain management.
